# A complete genome sequence of *Leifsonia* sp. McL0608, a soil bacterium from *Pinus radiata* forest

**DOI:** 10.1128/mra.00691-25

**Published:** 2025-10-21

**Authors:** Charlotte Armstrong, Mariann Eagle, Steven A. Wakelin

**Affiliations:** 1 Ecology and Environment, Scion, Bioeconomy Science Institute, Christchurch, New Zealand; California State University San Marcos, San Marcos, California, USA

**Keywords:** actinobacteria, forest soil, soil bacteria, bacterial genome, nanopore sequencing, genome annotation

## Abstract

*Leifsonia* sp. McL0608, isolated from *Pinus radiata* forest soil in New Zealand, has a complete 4.2 Mb circular genome assembled from Nanopore reads. Genome annotations identified 3,942 coding sequences and predicted biosynthetic pathways for alkanes, terpenes, and auxins.

## ANNOUNCEMENT

*Pinus radiata* D Don (Monterey pine) is one of the most widely planted tree species globally (1). Soils beneath *P. radiata* plantations support essential ecosystem services, such as nutrient cycling, carbon storage, and biodiversity (2). Actinobacteria comprise ~20% of the bacterial community associated with *P. radiata* roots and soils (3). Despite being widespread, most Actinobacteria are poorly described in terms of function and ecological roles (4). Incorporating annotated genomes of cultured isolates into reference databases is essential for improving functional interpretations of sequencing data.

*Leifsonia* sp. McL0608 was isolated from topsoil collected at 0–10 cm depth in a second rotation *P. radiata* plantation in New Zealand (43.4656 S 172.3992 E) in autumn 2022. The soil is classified as fluvial recent soil from alluvial floodplains; it is stony, well-draining, and nutrient poor (5). A 1:100 soil:water mix was shaken for 4 h then serially diluted to 10^−6^ g soil mL^−1^. Aliquots of 100 µL were spread on Reasoner’s 2A agar and incubated at room temperature under dark conditions until a single colony formed. After 1 week, a circular creamy colony was putatively identified via full-length 16S rRNA gene sequencing using 785F/907R primers (Macrogen; South Korea), showing similarity to *Diaminobutyricibacter tongyongensis* (GenBank accession NR_134019.1) after performing a BLASTn version 2.16.0 + search against the NCBI RefSeq 16S rRNA database, release number 229 (6).

DNA was extracted using the Wizard Genomic DNA Kit (Promega, USA) following the manufacturer’s gram-positive bacteria protocol. Genomic DNA was sequenced using long-read Oxford Nanopore Technology (ONT; UK) using a FLO-MIN114 R10.4.1 flow cell on a PromethION platform. DNA was prepared for sequencing using the Rapid Barcoding Kit V14 (SQK-RBK114.96) following the ONT DNA V14 protocol; no shearing or size selection was performed. “Super-accurate” base calling was performed using Guppy version 6.3.9 (7).

Sequencing ran for 68 h and generated 28,663 reads (average length 3,363 bp; max length 77,981 bp; N_50_ 8,698 bp). Quality control and trimming were performed using chopper version 0.8.0 (8) with a Q-score of 10; 57% of reads passed quality filtering. The genome was assembled with raven-assembler version 1.8.3 (9) and polished with medaka version 1.12.1 (10).

The genome consists of a single contig, with an N_50_ and a total size of 4,196,112 bp, GC content of 66.74%, and 21× coverage/depth. The circular nature of the genome was confirmed by Bandage version 0.8.1 (11), which showed a closed circular contig without dead ends or breaks. The genome was not rotated.

Genome annotation was performed using NCBI Prokaryotic Genome Annotation Pipeline version 6.7 (12) resulting in 3,942 CDSs (protein-coding sequence), 3 rRNA, 46 tRNA, and 1 tmRNA. Secondary metabolism was predicted using antiSMASH version 8.0 (13) and RAST (14), which includes genes for non-alpha poly-amino acid, ribosomally synthesized and post-translationally modified peptide products, type III polyketide synthase, terpene synthesis, auxin synthesis, and alkane biosynthesis.

Taxonomic placement of McL0608 was initially assigned to the genus *Diaminobutyricibacter* based on genome phylogeny using Genome Taxonomy Database version 10-RS226 (15). However, this genus has since been reclassified as a later heterotypic synonym of *Leifsonia* (16).

## Data Availability

The complete genome sequence is available on GeneBank under the accession number CP154826.1 and the Nanopore raw reads can be accessed under the SRA accession number SRX29315190. All predicated secondary metabolites generated by antiSMASH and RAST are publicly available on Figshare (https://doi.org/10.6084/m9.figshare.29178488).

## References

[1] Mead DJ. 2013. Sustainable management of *Pinus radiata* plantations. In FAO Forestry Paper. Vol. 170. Food and Agriculture Organization of the United Nations.

[2] Yao RT, Barry L, Wakelin S, Harrison D, Magnard L, Payn T. 2013. Planted forests, p 62–78. In Dymond J (ed), New Zealand ecosystem services: conditions and trends. Manaaki Whenua Press, Palmerston North, New Zealand.

[3] Addison SL, Smaill SJ, Garrett LG, Wakelin SA. 2019. Effects of forest harvest and fertiliser amendment on soil biodiversity and function can persist for decades. Soil Biology and Biochemistry 135:194–205. doi:10.1016/j.soilbio.2019.05.006

[4] Delgado-Baquerizo M, Oliverio AM, Brewer TE, Benavent-González A, Eldridge DJ, Bardgett RD, Maestre FT, Singh BK, Fierer N. 2018. A global atlas of the dominant bacteria found in soil. Science 359:320–325. doi:10.1126/science.aap9516 29348236

[5] Manaaki Whenua - Landcare Research. 2019. S-Map - New Zealand’s national digital soil map. Available from: 10.7931/L1WC7

[6] Camacho C, Coulouris G, Avagyan V, Ma N, Papadopoulos J, Bealer K, Madden TL. 2009. BLAST+: architecture and applications. BMC Bioinformatics 10:421. doi:10.1186/1471-2105-10-421 20003500 PMC2803857

[7] Oxford Nanopore Technologies. 2023 Guppy basecalling software. Available from: https://community.nanoporetech.com

[8] 2022. Chopper. Available from: https://github.com/wdecoster/chopper

[9] Vaser R, Šikić M. 2021. Time- and memory-efficient genome assembly with raven. Nat Comput Sci 1:332–336. doi:10.1038/s43588-021-00073-4 38217213

[10] Medaka. 2018. Oxford nanopore technologies. Available from: https://github.com/nanoporetech/medaka

[11] Wick RR, Schultz MB, Zobel J, Holt KE. 2015. Bandage: interactive visualization of de novo genome assemblies. Bioinformatics 31:3350–3352. doi:10.1093/bioinformatics/btv383 26099265 PMC4595904

[12] Tatusova T, DiCuccio M, Badretdin A, Chetvernin V, Nawrocki EP, Zaslavsky L, Lomsadze A, Pruitt KD, Borodovsky M, Ostell J. 2016. NCBI prokaryotic genome annotation pipeline. Nucleic Acids Res 44:6614–6624. doi:10.1093/nar/gkw569 27342282 PMC5001611

[13] Blin K, Shaw S, Vader L, Szenei J, Reitz ZL, Augustijn HE, Cediel-Becerra JDD, de Crécy-Lagard V, Koetsier RA, Williams SE, et al.. 2025. antiSMASH 8.0: extended gene cluster detection capabilities and analyses of chemistry, enzymology, and regulation. Nucleic Acids Res 53:W32–W38. doi:10.1093/nar/gkaf334 40276974 PMC12230676

[14] Brettin T, Davis JJ, Disz T, Edwards RA, Gerdes S, Olsen GJ, Olson R, Overbeek R, Parrello B, Pusch GD, Shukla M, Thomason JA, Stevens R, Vonstein V, Wattam AR, Xia F. 2015. RASTtk: a modular and extensible implementation of the RAST algorithm for building custom annotation pipelines and annotating batches of genomes. Sci Rep 5:8365. doi:10.1038/srep08365 25666585 PMC4322359

[15] Parks DH, Chuvochina M, Rinke C, Mussig AJ, Chaumeil PA, Hugenholtz P. 2022. GTDB: an ongoing census of bacterial and archaeal diversity through a phylogenetically consistent, rank normalized and complete genome-based taxonomy. Nucleic Acids Res 50:D785–D794. doi:10.1093/nar/gkab776 34520557 PMC8728215

[16] Gtari M, Boussoufa D, Ghodhbane-Gtari F, Sbissi I. 2025. Taxonomic rearrangement of Salinibacterium, Leifsonia, Diaminobutyricibacter, Antiquaquibacter, Homoserinimonas and Glaciibacter: refining genus boundaries and proposal of two new genera – Orlajensenia gen. nov. and Leifsonella gen. nov. Int J Syst Evol Microbiol 75. doi:10.1099/ijsem.0.006809 40531662

